# Homoarginine ameliorates diabetic nephropathy independent of nitric oxide synthase‐3

**DOI:** 10.14814/phy2.14766

**Published:** 2021-03-13

**Authors:** Michael D. Wetzel, Kristen Stanley, Soumya Maity, Muniswamy Madesh, Jean C. Bopassa, Alaa S. Awad

**Affiliations:** ^1^ Department of Medicine University of Texas Health Science Center at San Antonio San Antonio TX USA; ^2^ Department of Cellular and Integrative Physiology University of Texas Health Science Center at San Antonio San Antonio TX USA

**Keywords:** cardiac function, diabetic nephropathy, homoarginine, NOS3

## Abstract

Recently we showed that homoarginine supplementation confers kidney protection in diabetic mouse models. In this study we tested whether the protective effect of homoarginine is nitric oxide synthase‐3 (NOS3)‐independent in diabetic nephropathy (DN). Experiments were conducted in NOS3 deficient (NOS3^−/−^) mice and their wild type littermate using multiple low doses of vehicle or streptozotocin and treated with homoarginine via drinking water for 24 weeks. Homoarginine supplementation for 24 weeks in diabetic NOS3^−/−^ mice significantly attenuated albuminuria, increased blood urea nitrogen, histopathological changes and kidney fibrosis, kidney fibrotic markers, and kidney macrophage recruitment compared with vehicle‐treated diabetic NOS3^−/−^ mice. Furthermore, homoarginine supplementation restored kidney mitochondrial function following diabetes. Importantly, there were no significant changes in kidney NOS1 or NOS2 mRNA expression between all groups. In addition, homoarginine supplementation improved cardiac function and reduced cardiac fibrosis following diabetes. These data demonstrate that the protective effect of homoarginine is independent of NOS3, which will ultimately change our understanding of the mechanism(s) by which homoarginine induce renal and cardiac protection in DN. Homoarginine protective effect in DN could be mediated via improving mitochondrial function.

## Graphical abstract

Our results indicate that homoarginine mediates renal and cardiac protection in diabetic NOS3‐/‐ mice which will ultimately change our understanding of the mechanism(s) by which homoarginine ameliorate diabetic kidney injury. Homoarginine protective effect in diabetic nephropathy could be mediated via improving mitochondrial function.

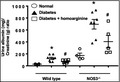

## 1 INTRODUCTION

Diabetes is a serious and increasingly prevalent cause of morbidity and mortality in the United States, with an estimated 12–14% of adults being diabetic, with even higher rates among ethnic minorities (Gao et al., 2016; Menke et al., 2015). Economic and productivity losses due to diabetic complications are over $245 billion and leads to physiological and psychological effects including systemic inflammation, renal and cardiac dysfunction, depression, and social anxiety (Jha et al., 2018; Menke et al., 2015; Moreno et al., 2018; Tao et al., 2015; Vanstone et al., 2015). Diabetic nephropathy (DN) is a chronic progressive disorder leading to a rapid decline and end‐stage kidney failure. Over the last decade, the incidence of kidney failure due to diabetes has doubled. Traditional therapies, including blood pressure and glucose control and other lifestyle changes, have only been modestly successful in delaying the progression of renal failure. Thus, it is important to identify the mechanisms involved in the development and progression of diabetic kidney disease.

Homoarginine, an endogenous arginine analog, not involved in protein synthesis, has emerged as a new player in health and disease (Pilz et al., 2015). Homoarginine is synthesized by mitochondrial arginine:glycine amidinotransferase (AGAT) in kidneys using l‐arginine and l‐lysine as substrates (Choe et al., 2013; Ryan & Wells, 1964), and catabolized using alanine:glyoxylate aminotransferase 2 (AGXT2) (Rodionov et al., 2016). Plasma concentrations of homoarginine are inversely correlated with the risk of cardiovascular disease and overall mortality (Jud et al., 2018; Marz et al., 2010), increased risk for fatal strokes (Haghikia et al., 2017), congestive heart failure and left ventricular hypertrophy (Atzler et al., ,^2013^, ^2017^; Bahls et al., 2018; Pilz, Edelmann, et al., 2014), aging (Atzler, Schwedhelm, et al., 2014; Marz et al., 2010), smoking (Atzler, Gore, et al., 2014; Sobczak et al., 2014; Vogl et al., 2015; Zwan et al., 2013), body mass index (Atzler, Gore, et al., 2014; Marz et al., 2010; Pilz, Teerlink, et al., 2014), and pregnancy (Valtonen et al., 2008). Low homoarginine levels act as an indicator of cardiac dysfunction, with associated high levels of asymmetric dimethylarginine (ADMA) (Atzler, Schwedhelm, et al., 2014; Jud et al., 2018; Kayacelebi et al., 2014; Pilz et al., 2015; Vogl et al., 2015). The effects of low homoarginine on nitric oxide (NO) production/action have been linked with increased risk of stroke and atherosclerosis (Haghikia et al., 2017). Endothelial nitric oxide synthase (NOS3) is constitutively expressed in endothelial cells, and it functions to adjust intracellular NO levels in response to calcium levels (Zanchi et al., 2000). Homoarginine may contribute to NO synthesis by serving as a substrate for NOS because homoarginine, even with its weaker affinity to NOS as compared to arginine, can be metabolized to NO and homocitrulline (Drechsler et al., 2011). In the kidney, low homoarginine is a strong risk factor for sudden cardiac death and death due to heart failure in hemodialysis patients (Drechsler et al., 2011). Furthermore, low homoarginine levels are associated with increased cardiovascular risk in renal transplant patients (Kayacelebi et al., 2017). In both animal (Banchaabouchi et al., 2001; Tofuku et al., 1985) and human (Drechsler et al., 2013) with chronic kidney injuries, homoarginine levels are reduced and low plasma homoarginine levels were significantly related to reduced glomerular filtration rate (GFR) and proteinuria (Ravani et al., 2013). Although epidemiological data showed that decreased homoarginine levels were associated with increased mortality in kidney and cardiovascular disease (Drechsler et al., 2015; Jud et al., 2018; Marz et al., 2010; Ravani et al., 2013), the direct physiologic mechanism(s) of homoarginine remain unclear. We previously showed that homoarginine is an effective treatment for DN (Wetzel et al., 2019). However, the mechanism of homoarginine action in kidney and cardiac dysfunction is not known.

In the current study, we tested the hypothesis that the protective effect of homoarginine in renal and cardiac functions in diabetes is independent of NOS3. Our results indicate that homoarginine mediates renal and cardiac protection in diabetic NOS3^−/−^ mice which will ultimately change our understanding of the mechanism(s) by which homoarginine ameliorate diabetic kidney injury. The protective effect of homoarginine in DN could be mediated via improving mitochondrial function.

## 2 MATERIALS AND METHODS

### 2.1 Diabetic mouse model

All animal experiments were performed in male 6‐week‐old C57BL/6J or NOS3^−/−^ mice (The Jackson Laboratory, Bar Harbor, ME). Mice were kept at 23°C and a 12:12 light–dark cycle with free access to standard chow and water. Diabetes was induced by intraperitoneal injection of 50 mg/kg streptozotocin (STZ) or saline control following a 6‐h fast for 5 consecutive days. All procedures were done in the morning throughout the study. Blood glucose levels were measured from non‐fasting mice using a Contour Next EZ glucometer with Contour Next strips from mice tail vein, with diabetic mice being defined as having a blood glucose level over 350 mg/dl. l‐Homoarginine (Sigma, St. Louis MO, Cat #: H1007) was supplemented in drinking water at a concentration of 50 mg/L as previously described (Wetzel et al., 2019) and replaced every 4 days. At the end of the study (24 weeks), 24‐h urine samples were collected, mice were euthanized using an intraperitoneal injection of ketamine/xylazine cocktail; plasma was obtained by cardiac puncture, and kidneys and hearts were removed for further studies. For urine collections mice were placed individually in metabolic cages for 16 h with free access to drinking water, and urine was collected in collecting tubes containing 200 µl of mineral oil that prevents urine evaporation. Blood was collected by cardiac puncture and centrifuged at 3000 *g* for 3 min at 4°C to separate plasma. Following collection all samples were stored at −80°C until analysis. The number of mice per group is given in Table 1. Samples derived from *Ins2^Akita^* mice were obtained as previously described (You et al., 2015). All the animal experiments were approved by the University of Texas Health Science Center at San Antonio Institutional Animal Care and Use Committee.

**TABLE 1:** Mouse characteristics

	WT	WT + Diabetes	WT + Diabetes + HA	NOS3^−/−^	NOS3^−/−^ + Diabetes	NOS3^−/−^ + Diabetes + HA
No. per group	5	8	8	4	8	5
Body weight: Baseline (g)	21.8 ± 0.4	23.4 ± 0.9	20.3 ± 0.4	19.5 ± 0.9	21.1 ± 0.7	21.6 ± 0.5
Body weight: % change from baseline	140 ± 6	112 ± 3^a^	123 ± 6^a^	147 ± 5	101 ± 7^b^, ^c^	122 ± 7^c^
Blood glucose (mg/dl)	160 ± 8	463 ± 73^a^	429 ± 62^a^	151 ± 13	523 ± 51^b^, ^c^	386 ± 58^b^, ^c^
Kidney/body weight ratio	0.63 ± 0.02	0.68 ± 0.02	0.66 ± 0.05	0.58 ± 0.03	0.74 ± 0.05^b^, ^c^	0.65 ± 0.04
Heart/body weight ratio	0.53 ± 0.02	0.53 ± 0.02	0.51 ± 0.03	0.50 ± 0.02	0.57 ± 0.02	0.46 ± 0.03^c^
SBP (mmMg)	122 ± 8	114 ± 10	117 ± 7	138 ± 10	170 ± 5^b^, ^c^	150 ± 7
Water Intake: Baseline (ml)	5.5 ± 0.6	4.2 ± 0.6	2.7 ± 0.6	3.0 ± 0.4	1.1 ± 0.3	1.1 ± 0.2
Water Intake: % change from baseline	208 ± 46	202 ± 17	242 ± 51	334 ± 68	519 ± 129	581 ± 157

Mice were injected with streptozotocin for 5 consecutive days, then treated with or without homoarginine in drinking water for 24 weeks. Data are presented as mean ± SEM.

Baseline measurement were obtained at week zero.

Abbreviations: HA, homoarginine; SBP, systolic blood pressure; WT, wild type.

^a^ *p*<0.05 versus WT.

^b^ *p*<0.05 versus NOS3^−/−^.

^c^ *p*<0.05 versus NOS3^−/−^ + Diabetes.

### 2.2 Immunohistochemistry

Mouse kidney tissues were fixed in 10% formalin and embedded in paraffin, and 3‐μm sections were cut. Immunohistochemistry was performed on paraffin‐embedded sections with anti‐mouse Mac‐2 antibody (clone M3/38; Cedarlane, Burlington, NC) as previously described (You et al., 2013, 2014). Images were taken with an Olympus BX60 microscope and Olympus Q‐Color3 digital camera using Q‐capture Pro 7 image software. The number of glomerular macrophages was counted in 20 glomeruli/section (no. of macrophages in glomeruli divided by the no. of glomeruli) in a blinded fashion under 40× magnification and averaged as described previously (Morris et al., 2011; Wetzel et al., 2019; You et al., 2013, 2014).

### 2.3 Renal and cardiac histopathology

Kidneys and hearts were fixed in 4% paraformaldehyde, embedded in paraffin, and 5‐μm sections were cut. Sections were stained with Masson's trichrome or periodic acid‐Schiff (PAS) stain, examined in a blinded manner, and scores were averaged. To determine percent area fibrosis, Masson's trichrome pictures were obtained at 10x and analyzed in ImageJ to measure percent area of fibrosis. Representative images were taken with an Olympus BX60 microscope and Olympus Q‐Color3 digital camera using Q‐capture Pro 7 image software. Glomerular characteristics were measured using Bioquant Osteo software (Bioquant Image Analysis Corporation, Nashville, TN), and percent injury index was calculated as described previously (Mohan et al., 2008). For each kidney, we obtained 12 images and analyzed a glomerulus for each field to be averaged for each animal for statistical analysis.

### 2.4 Analytical methodology

Urine albumin concentration was measured by ELISA using an Albuwell M kit (Exocell, Philadelphia, PA) as described previously (Awad, You, Gao, Gvritishvili, et al., 2015). Urine creatinine was measured using Diazyme Creatinine Assay (Diazyme Laboratories, Poway, CA) (Awad et al., 2011; Awad, You, Gao, Cooper, et al., 2015). Blood urea nitrogen (BUN) was determined using a QuantiChrom urea assay kit (BioAssay Systems, Hayward, CA) as previously described (You et al., 2017). Mouse systolic pressures were measured using CODA Non‐invasive Blood Pressure system (Kent Scientific Corporation, Torrington CT) as previously described (Awad, You, Gao, Gvritishvili, et al., 2015). Mice were acclimated for 10 min at 26°C before readings began. Readings were taken at the same time of day for all groups to prevent any diurnal variations.

### 2.5 Cardiac function measurements

Serial B‐Mode and M‐Mode echocardiography was performed using a Vevo 2100 Imaging Platform (VisualSonics, Toronto, Canada) using a 30‐MHz linear transducer to accurately monitor cardiac hemodynamic parameters and assess heart structure of mice after 22 weeks of homoarginine treatment. The left ventricular ejection fraction (EF) and left ventricle fractional shrinkage (FS) were quantified using M‐mode as previously described (Iorga et al., 2016, 2018).

### 2.6 RNA isolation and real‐time PCR

RNA was isolated by Trizol extraction from whole kidney sections and reverse‐transcribed to cDNA using the Bio‐Rad (Hercules, CA) iScript cDNA synthesis kit. Real‐time PCR was performed on a CFX384 real‐time system (Bio‐Rad, Hercules, CA) using Taqman primers for mouse MCU (Mm0116873_m1), fibronectin (Mm01256744_m1), tumor growth factor β (TGFβ, Mm01178820_m1), NOS1 (Mm00435175_m1), NOS2 (Mm00440502_m1), and Rn18S (Mm03928990_g1) (Thermo Fisher, Waltham, MA). Ct values were normalized to 18S and compared with NOS3^−/−^ samples. Primers for smooth muscle actin (FW: 5′‐CTGCCGTTTTCCCCCTTCCTG‐3′, RV: 5′‐TTGCTTCCTCCTCCTTTG‐3′), E‐cadherin (FW: 5′‐TGAGTGTGTGGGTGCTGA‐3′, RV: 5′‐CGGTTTCAATGGCTTACCTTTTCC‐3′), and β‐actin (FW: 5′‐GCTGGTTGTGTAAGGTAAGGTGTGC‐3′, RV: 5′‐GAGGGGGTTGAGGTGTTGAGG‐3′) were obtained from Integrated DNA Technologies (Coralville, IA). cDNA samples were analyzed using SYBR Green Master Mix (Thermo Scientific, Rockford IL), as previously described (Morris et al., 2011), and normalized to β‐actin for comparison to NOS3^−/−^ samples.

### 2.7 Western blotting

Kidney tissue was homogenized in RIPA buffer containing 0.1% Triton X‐100 supplemented with protease inhibitors (Roche Diagnostics, Indianapolis, IN) and cleared by centrifugation at 10,000 *g* for 10 min at 4°C, and supernatant was collected. Protein concentration was determined by Bicinchonic Acid (BCA) assay (Thermo Scientific, Waltham, MA). A sample of 50 μg of kidney lysate was separated on 4–12% Bis–Tris gel (Life Technologies, Carlsbad, CA) and transferred onto PVDF membranes before blocking with 5% dry milk. Western blots were performed using the following antibodies: MiCU1 (0.4 µg/ml; Sigma‐Aldrich, St Louis, MO) and β‐actin (1:1000; Cell Signaling, Danvers, MA) antibodies. Western blots were quantitated using ImageJ software (NIH, Bethesda, MD) and normalized to β‐actin protein expression.

### 2.8 Statistical analysis

Comparisons between all groups were conducted using GraphPad Prism software (version 7.04, San Diego, CA). Results are expressed as mean ± SEM. One‐way ANOVA followed by Fisher's least significance difference test was used to compare significance between groups. A value of *p* < 0.05 represented significant difference.

## 3 RESULTS

### 3.1 Mouse characteristics

Diabetic wild type and NOS3^−/−^ mice had significantly less body weight compared with non‐diabetic mice after 24 weeks, which was significantly increased by homoarginine in diabetic NOS3^−/−^ mice. Blood glucose was higher in all diabetic wild type and NOS3^−/−^ mice. Kidney/body weight ratio was significantly higher in diabetic NOS3^−/−^ mice, while homoarginine treatment significantly reduced cardiac/body weight ratio in diabetic NOS3^−/−^ mice. Systolic blood pressure was significantly increased in NOS3^−/−^ mice compared with wild type and showed a trend of reduction; albeit not significant by homoarginine treatment in diabetic NOS3^−/−^ mice. (Table 1).

### 3.2 Homoarginine reduces albuminuria in diabetic NOS3^−/−^ mice

**FIGURE 1:**
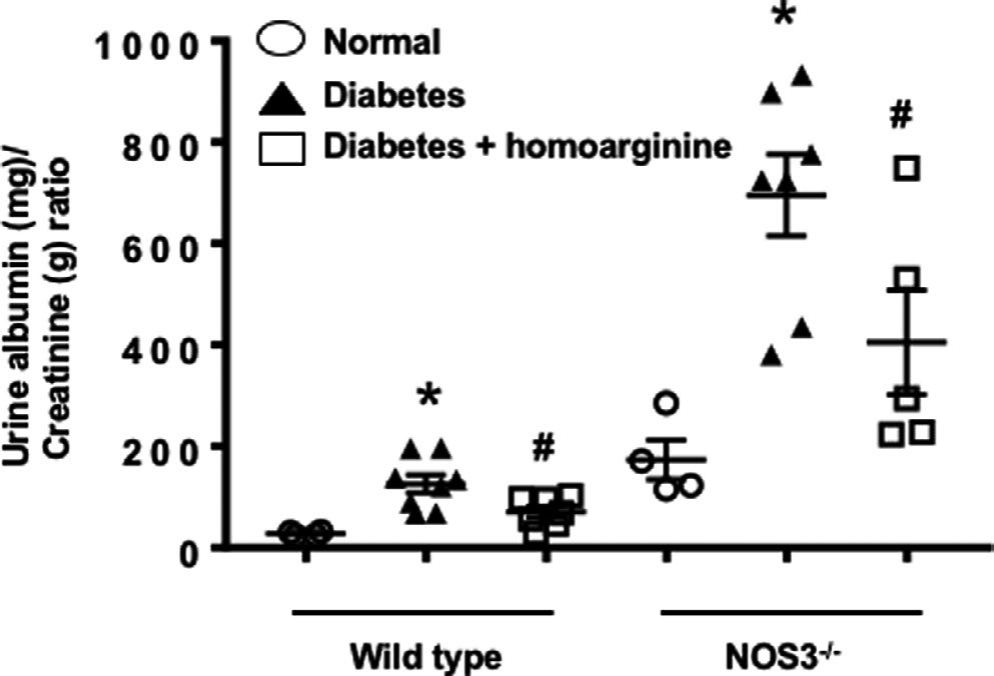
Homoarginine reduces albuminuria in diabetic mice. Urine was collected from wild type and NOS3^−/−^ mice after 24 weeks of diabetes and analyzed for albumin/creatinine ratio. Data are presented as mean ± SEM. *p < 0.05 versus normal, ^#^ *p* < 0.05 versus diabetes.

Diabetes significantly increased albuminuria in both wild type and NOS3^−/−^ mice compared with normal, an effect significantly reduced by homoarginine treatment (Figure 1). Since the results observed in wild type mice were consistent with our previous study (Wetzel et al., 2019) about the protective effect of homoarginine in DN, we only analyzed NOS3^−/−^ mice for the remainder of this study.

### 3.3 Homoarginine reduces plasma BUN levels in diabetic NOS3^−/−^ mice

**FIGURE 2:**
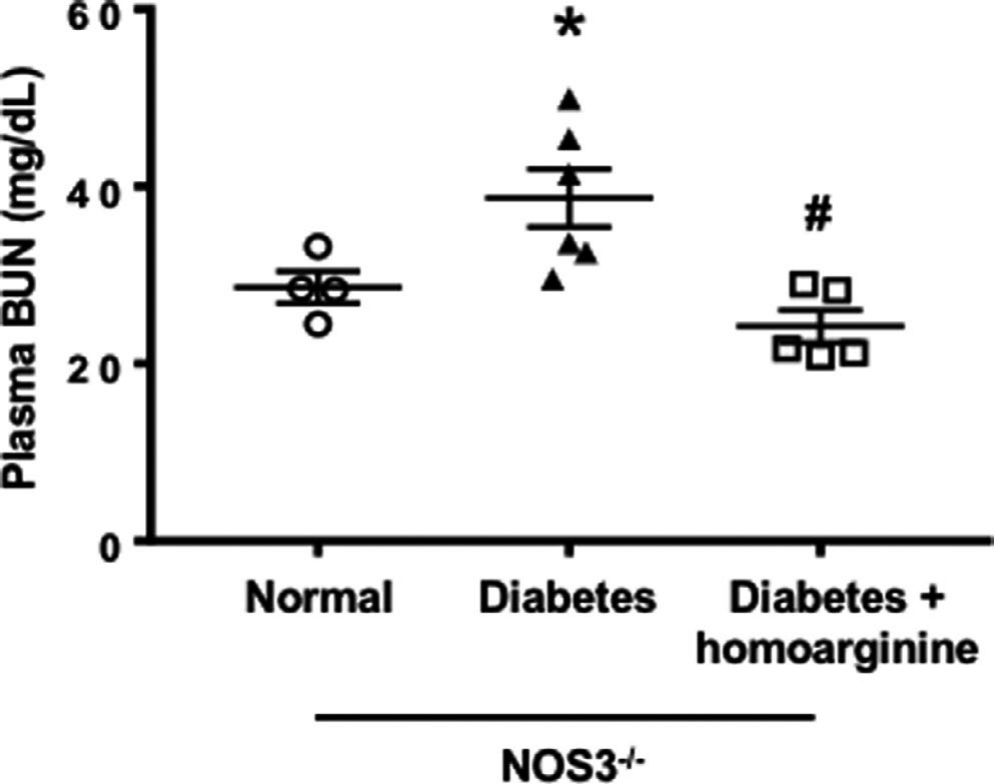
Homoarginine reduces plasma BUN in diabetic NOS3^−/−^ mice. Plasma BUN was determined in NOS3^−/−^ mice after 24 weeks of diabetes. Data are presented as mean ± SEM. *p < 0.05 versus normal, ^#^ *p* < 0.05 versus diabetes.

We next evaluated the effects of homoarginine administration on plasma BUN levels in NOS3^−/−^ mice. Plasma BUN levels were increased in diabetic mice and reduced by homoarginine treatment (Figure 2).

### 3.4 NOS1 and NOS2 expressions are not altered in diabetic NOS3^−/−^ mice

**FIGURE 3:**
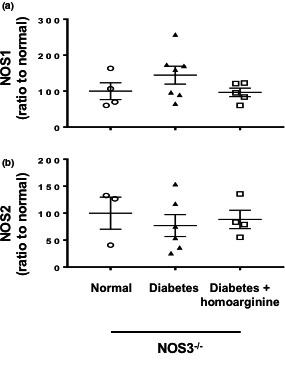
Expression of NOS1 and NOS2 are not altered in diabetic NOS3^−/−^ mice. RT‐PCR was performed on cDNA extracted from kidney samples of mice and analyzed for (a) NOS1 and (b) NOS2. Data are presented as mean ± SEM.

Next we determined if diabetes and/or homoarginine altered the expression of other NOS isoforms in kidneys from NOS3 ^−/−^ mice. Our results show that neither diabetes nor homoarginine affected NOS1 or NOS2 gene expression in NOS3^−/−^ mice (Figure 3).

### 3.5 Homoarginine reduces glomerular pathology and fibrosis in diabetic NOS3^−/−^ mice

**FIGURE 4:**
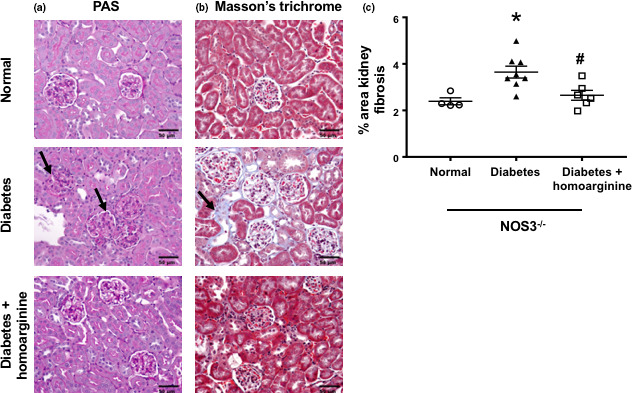
Homoarginine reduces glomerular pathology and fibrosis in diabetic NOS3^−/−^ mice. (a) Paraffin‐embedded mouse kidney sections were subjected to periodic acid‐Schiff (PAS) staining. (b) Kidney sections were fixed in formaldehyde and stained with Masson's Trichrome. (c) Images from b were quantified for percent area fibrosis using ImageJ. Data are presented as mean ± SEM. *p < 0.05 versus normal, ^#^ *p* < 0.05 versus diabetes. Pictures taken at 40× magnification. Black arrow: area of fibrosis.

Analysis of PAS‐stained glomerular sections showed that diabetic NOS3^−/−^ mice were associated with increased glomerular volume, glomerular cellularity, glomerular basement membrane (GBM) thickness, and glomerular injury index compared with non‐diabetic NOS3^−/−^ mice; an effect significantly reduced using homoarginine treatment (Table 2 and Figure 4a). Furthermore, diabetic NOS3^−/−^ mice displayed an increased percent of fibrosis (using ImageJ) in Masson's trichrome (Figure 4b,c)‐stained sections 24 weeks after diabetes, which was reduced by treatment with homoarginine.

**TABLE 2:** Homoarginine reduces glomerular injury index in NOS3^−/−^ mice.

	NOS3^−/−^	NOS3^−/−^ + Diabetes	NOS3^−/−^ + Diabetes + HA
Glomerular volume/µM^2^	3838 ± 299	4711±269^a^	4356 ± 169
Cellularity (cgs)	18.7 ± 1.2	29.4 ± 0.7^a^	23.1 ± 0.6^a^, ^b^
Injury index %	4.2 ± 2.4	22.9 ± 4.1^a^	8.3 ± 4.1^b^
GBM thickness (µM)	0.34 ± 0.01	0.50 ± 0.02^a^	0.39 ± 0.02^b^

Note: PAS‐stained sections were analyzed using Bioquant Osteo for the indicated parameters of glomerular pathology.

HA, homoarginine; GBM, glomerular basement membrane.

^a^ *p* < 0.05 versus NOS3^−/−^.

^b^ *p* < 0.05 versus NOS3^−/−^ + Diabetes.

### 3.6 Homoarginine reduces kidney fibrotic markers in diabetic NOS3^−/−^ mice

**FIGURE 5:**
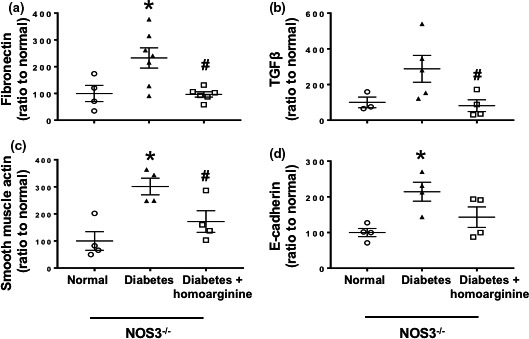
Homoarginine reduces fibrotic markers in diabetic NOS3^−/−^ mice. RT‐PCR was performed on cDNA extracted from kidney samples of mice and analyzed for (a) fibronectin, (b) TGFβ, (c) smooth muscle actin, (d) E‐cadherin. Data are presented as mean ± SEM. *p < 0.05 versus normal, ^#^ *p* < 0.05 versus diabetes.

Real‐time PCR analysis show marked increases in kidney fibronectin (Figure 5a), TGFβ (Figure 5b), smooth muscle actin (Figure 5c), and E‐cadherin (Figure 5d) following diabetes in NOS3^−/−^ mice. Homoarginine supplementation significantly blunted the increase in these fibrosis markers.

### 3.7 Homoarginine reduces glomerular macrophage infiltration in diabetic NOS3^−/−^ mice

**FIGURE 6:**
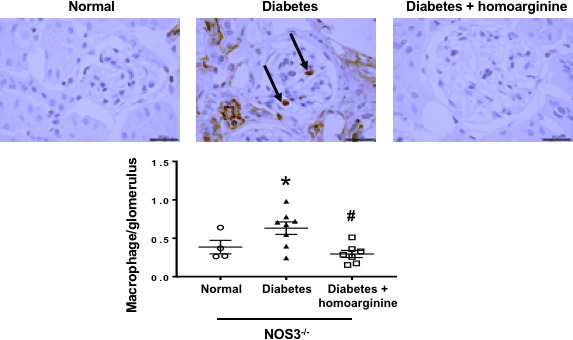
Homoarginine reduces glomerular macrophage infiltration in diabetic NOS3^−/−^ mice. Glomerular macrophage recruitment was visualized by immunohistochemical staining using Mac‐2 antibody. The number of glomerular macrophages was counted in 40 glomeruli per section (number of macrophages in glomeruli divided by the number of glomeruli) in blinded fashion under 40× magnification and averaged. Data are presented as mean ± SEM. *p < 0.05 versus normal, ^#^ *p* < 0.05 versus diabetes. Pictures taken at 100× magnification. Black arrow: macrophage.

Diabetic NOS3^−/−^ mice displayed a marked increase in infiltrating kidney macrophages following diabetes (Figure 6) compared with non‐diabetic mice. Homoarginine treatment significantly reduced macrophage infiltration 24 weeks after diabetes.

### 3.8 Homoarginine reduces cardiac fibrosis and improves cardiac function in diabetic NOS3^−/−^ mice

**FIGURE 7:**
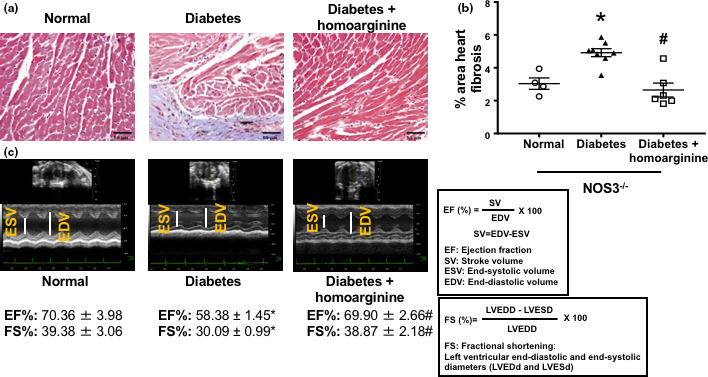
Homoarginine reduces cardiac fibrosis and improves cardiac function in diabetic NOS3^−/−^ mice. (a) Masson's Trichrome staining of heart sections. Pictures taken at 40× magnification. (b) Fibrosis scoring of Masson's Trichrome heart sections from A using ImageJ. (c) Vevo 2100 Imaging Platform was used to image mouse hearts and determine percentage of left ventricular ejection fraction (EF) and fractional shrinking (FS). Data are presented as mean ± SEM. *p < 0.05 versus normal, ^#^ *p* < 0.05 versus diabetes.

Diabetic NOS3^−/−^ mice displayed an increased percent of fibrosis (using ImageJ) in Masson's trichrome (Figure 7a,b)‐stained sections 24 weeks after diabetes, which was reduced by treatment with homoarginine. Furthermore, diabetic NOS3^−/−^ mice had a significant reduction in ejection fraction (EF) and fractional shrinking (FS) (Figure 7c–e) compared with non‐diabetic controls at 22 weeks of diabetes, an effect significantly restored using homoarginine treatment.

### 3.9 Homoarginine restores MCU complex and its regulators in diabetic Ins2^Akita^ mice

**FIGURE 8:**
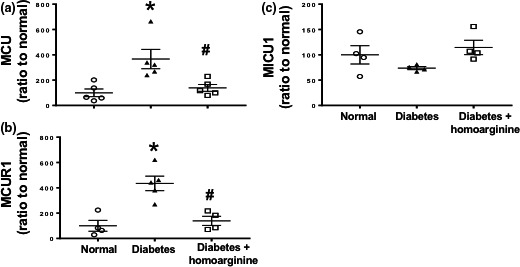
Homoarginine restores kidney MCU complex and its regulators in diabetic Ins2^Akita^ mice. RT‐PCR was performed on cDNA extracted from kidney samples derived from our previous publication (Wetzel et al., 2019) and were analyzed for (a) MCU complex, (b) MCU positive regulator, MCUR1, and (c) MCU negative regulator, MICU1. Data are presented as mean ± SEM. *p < 0.05 versus normal, ^#^ *p* < 0.05 versus diabetes.

Kidney samples derived from our previous publication (Wetzel et al., 2019) were analyzed for MCU complex and its regulators. Diabetic mice had a significant increase in kidney MCU and its positive regulator, MCUR1 expression, along with a reduction in kidney MCU negative regulator, MICU1 expression (Figure 8), compared with non‐diabetic controls at 18 weeks of age in Ins2^Akita^ mice, whereas diabetic mice treated with homoarginine supplementation restored MCU complex and its regulators to normal levels.

### 3.10 Homoarginine restores MCU complex and its regulators in diabetic NOS3^−/−^ mice

**FIGURE 9:**
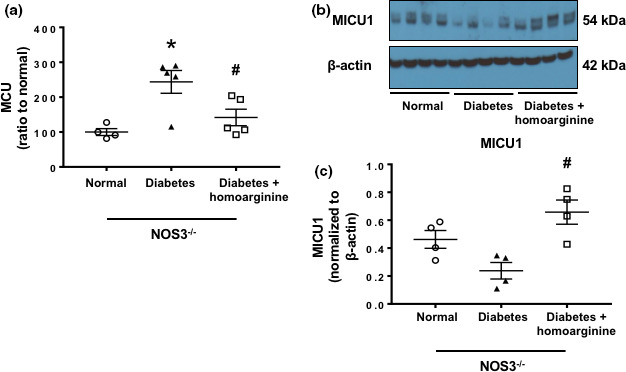
Homoarginine restores MCU complex and its regulators in diabetic NOS3^−/−^ mice. (a) RT‐PCR analysis of MCU gene expression from mouse kidneys. (b) Protein was isolated from kidneys of NOS3^−/−^ mice. Western blotted for MICU1 with β‐actin as protein‐loading control. (c) Quantification of MICU1 protein expression. Data are presented as mean ± SEM. *p < 0.05 versus normal, ^#^ *p* < 0.05 versus diabetes.

Diabetic NOS3^−/−^ mice had a significant increase in kidney MCU (Figure 9a) along with a significant reduction in its negative regulator, MICU1 expression (Figure 9b,c), compared with non‐diabetic controls after 24 weeks of diabetes, whereas diabetic NOS3^−/−^ mice treated with homoarginine supplementation restored MCU complex and its negative regulator to normal levels.

## 4 DISCUSSION

Diabetic nephropathy remains an important and unresolved complication of diabetes. Our previous publication demonstrated that homoarginine supplementation confers kidney protection in diabetic mouse models (Wetzel et al., 2019). However, the exact mechanism by which homoarginine mediates these effects was not clear. The current study shows that homoarginine supplementation mediates renal tissue protection in DN independent of NOS3 which will ultimately change our understanding of the mechanism(s) by which homoarginine induce renal and cardiac protection in DN. The protective effect of homoarginine in DN could be mediated via improving mitochondrial function.

Endothelial dysfunction, characterized by reduced bioavailability of NO, and increased oxidative stress is a hallmark characteristic in diabetes (Creager et al., 2003) and DN (Goligorsky et al., 2001). The role of NOS3 expression and action in DN is controversial; therefore although low or lack of NOS3 has been shown to exacerbate DN (Wang et al., 2011; Zhao et al., 2006), a recent publication (Natarajan et al., 2019) showed that overexpression of NOS3 in Ins2^Akita^ mice exacerbates DN probably via increased NOS3 uncoupling and oxidative stress rather than by increased renal NO production/action. Because homoarginine could be a substrate for NOS by increasing l‐arginine (Hecker et al., 1991; Moali et al., 1998), it could also mediate its effect via NO action/production. However, our previous publication showed that oral arginine supplementation surprisingly did not prevent or reduce any markers of renal injury in diabetic mouse model, despite successfully increasing arginine levels in plasma and kidney (You et al., 2014). Direct interaction between homoarginine levels and NO production is controversial. Homoarginine could reduce NO synthesis from arginine by (a) competition of arginine and homoarginine for cellular uptake by cationic amino acid transporters (CAT) (Chafai et al., 2017; Hagos et al., 2006); (b) by trans‐stimulation of CAT, which in turn could increase cellular efflux of arginine; and (c) by competition of homoarginine and arginine for NOS (Pilz et al., 2015), although homoarginine is a less efficient substrate for this enzyme (Moali et al., 1998; Zwan et al., 2013). Although homoarginine may increase arginine availability and thus NO synthesis by inhibition of arginase, this is unlikely because of the weak affinity of homoarginine for arginase (Marz et al., 2010). Nonetheless, homoarginine may contribute to NO synthesis by serving as a substrate for NOS because homoarginine, even with its weaker affinity to NOS as compared with arginine, can be metabolized to NO and homocitrulline (Drechsler et al., 2011).

To examine the mechanism of homoarginine's protective effect in DN, we investigated the effects of homoarginine supplementation on kidney dysfunction, glomerular histopathological changes, and macrophage recruitment in NOS3^−/−^ and their wild type littermate diabetic mouse model. Homoarginine supplementation significantly ameliorated diabetic albuminuria in diabetic wild type and NOS3^−/−^ mice, confirming our previous finding (Wetzel et al., 2019) and was associated with reduced glomerular pathology and kidney fibrotic markers expression in diabetic NOS3^−/−^ mice. Taken together, our results indicate a direct role of homoarginine's protective effect in DN and support the concept that homoarginine mediates renal tissue protection independent of NOS3.

Both cardiovascular morbidity and mortality are increased in patients with DN. In the heart, lower homoarginine levels are related to increased left ventricular thickening, lower EF, and higher NTproBNP (Bahls et al., 2018). Inversely, homoarginine supplementation preserve cardiac function in murine model of post‐myocardial infarction heart failure (Atzler et al., 2017). In the current study, we also demonstrate that the protective effect of homoarginine to improve cardiac function and reduce cardiac fibrosis is independent of NOS3 expression in a similar way to its effect on the kidney. We cannot however exclude the hemodynamic effect of homoarginine in DN given its trend to reduce blood pressure; albeit not significant.

The mechanism of homoarginine protective effect appears to be linked to modulations of mitochondrial function regulated by MCU complex and its regulators. Mitochondrial dysfunction plays a pivotal role in renal injury (Brooks et al., 2009; Coughlan & Sharma, 2016; Hallan & Sharma, 2016; Long et al., 2016; Sharma, 2015; Sun et al., 2008; Wang et al., 2012; You et al., 2016; Yu et al., 2006; Zhan et al., 2013). In the kidney, endothelial cells are especially vulnerable to mitochondrial dysfunction (Hong et al., 2012) and contributes to oxidative stress, persistent energy depletion, impairment of energy‐dependent repair mechanisms, and cell death in kidney injury (Funk et al., 2010; Funk & Schnellmann, 2012). MCU is a multimeric complex involved in rapid Ca^2+^ uptake in mitochondria and has been shown to be dysregulated in cardiac pathology (Liao et al., 2015; Luongo et al., 2015; Nemani et al., 2018; Tomar et al., 2019; Woods et al., 2019). Mitochondrial Ca^2+^ uptake mediated by MCU complex plays a critical role in signal transduction, bioenergetics, and cell death, and its dysregulation is linked to several human diseases. Although podocyte apoptosis has been linked to MCU dysregulation (Xu et al., 2018; Yuan et al., 2017), the role of MCU complex and its interaction with homoarginine supplementation in DN are not known. Our current data in diabetic mice demonstrated that homoarginine administration significantly reduced the expression of MCU, increased the MCU‐negative regulator (MICU1), and reduced the MCU‐positive regulator (MCUR1) in DN, and this effect is independent of NOS3 expression. Therefore, we speculate that there is an association between mitochondrial Ca^2+^ signaling and adverse clinical outcomes in DN. Normalization of mitochondrial function is necessary to restore oxidative metabolism during DN. Additional studies are needed to confirm the direct effect of homoarginine on mitochondrial function.

In conclusion, our study demonstrates for the first time that the mechanism of renal and cardiac tissue protection using homoarginine supplementation in DN is independent of NOS3, and it is likely mediated by improving mitochondrial dysfunction. Results of our study may ultimately result in novel therapeutic interventions designed to augment homoarginine in the treatment of DN.

## DISCLOSURE

The authors declare no conflicts of interest, financial, or otherwise.

## AUTHOR CONTRIBUTIONS

AA designed research studies. MW, KS, and SM conducted experiments and acquired and analyzed data. AA, JB, and MM analyzed data. MW and AA wrote manuscript. MW, KS, SM, MM, JB, and AA reviewed and edited manuscript.
